# Supplementary material for: The virtual microbiome: A computational framework to evaluate microbiome analyses
Source: PLoS One. 2023 Feb 8;18(2):e0280391. doi: 10.1371/journal.pone.0280391 (PMC9907852; doi:10.1371/journal.pone.0280391)
Supplement: Attachment — Submitted filename: Response to reviewers review 2.pdf [file pone.0280391.s004.pdf]

Please find below a point-by-point response (in blue) to the questions posed by the Reviewers (in black).

Reviewer #1: In the revised version of the manuscript, the authors made a remarkable effort to show the generality of their results by reproducing some parts of the analysis with a different taxonomic profiling tool (Kraken). Some obscure claims regarding unidentified taxa have been removed and the origin of false positives is now clearly explained. Overall, these changes notably improved the quality of the manuscript and the robustness of its conclusions.

We thank the Reviewer for his/her positive comments and for his/her contribution to the improvement of this article.

I appreciate the authors' effort to better describe the algorithm that produces virtual microbiomes. I still do not understand why they did not release a public, functional implementation of their code, although I leave it to the Editor to decide whether that is a requisite for publication. Anyway, the pseudocode included in the methods section is still incomplete: there are 4 subroutines in EcologyGeneration that are not proprely described. Furthermore, I could not find the parameter values (mu, sigma, shape, scale) used to generate the abundance matrices.

We forgot to add the link to the public repository where the code is available (https://github.com/bserranoanton/Virtual-microbiome). I apologize for this error. We have included it in the revised version of the text. In this site it is also possible to access the values of the parameters used to generate each virtual microbiome.

"The full implementation of the algorithm can be found at the following link https://github.com/bserranoanton/Virtual-microbiome. In addition, information on all the parameters used for the generation of the abundance matrices of the virtual microbiomes can also be accessed." (lines 139-142).

I understand that, from the authors' perspective, it is hard to let go of a fancy word like "virtualome", which is certainly catchy. However, I must insist that it is nonsense. There are already many "omes" in the recent biological literature, perhaps too many, but at least most of them are self-explanatory and semantically coherent. Genomes, proteomes, transcriptomes, interactomes, and microbiomes (to name a few) clearly refer to sets of genes, proteins, transcripts, interactions, and microbes, respectively. Accordingly, virtualomes would be sets of some kind of virtual entities. What does that mean? I could think of something like a set of virtual environments in a computing context, although that would be quite a stretch. However, the authors use the term to refer to a virtual microbiome (I guess that the rationale is to combine VIRTUAL with microbiOME). From a semantic perspective, that is nonsense. Following that rule, it could also mean virtual proteome, virtual interactome, virtual transcriptome, virtual metabolome... Or none of that. It is perhaps a funny word to describe the tool in an informal setting, among colleagues or perhaps as a joke in a conference if you want to get the attention of the audience. But I hardly see how it would be an acceptable (that is, unambiguous and semantically coherent) scientific term.

The Reviewer has provided solid arguments against the use of the word "virtualome". We have changed it accordingly throughout the text, including the title, and in the corresponding figures.

Some typos:

239: "does to distinguish" -> "does NOT distinguish"
325: "These unevenness in the relative abundance implies". Remove "These"

Corrected.

Reviewer #2: In this last version, the authors addressed all the concerns raised by the reviewers, extending certain sections and clarifying important aspects of the actual limitations of the metagenomics analysis. In particular, the new analysis performed using the Kraken2 well supported the results and conclusions of the manuscripts.

We thank the Reviewer for his/her positive evaluation of this work.
